# Supplementary material for: Multi-group analysis of grooming network position in a highly social primate
Source: PLoS One. 2023 Apr 26;18(4):e0284361. doi: 10.1371/journal.pone.0284361 (PMC10132689; doi:10.1371/journal.pone.0284361)
Supplement: S2 File — (DOCX) [file pone.0284361.s002.docx]

# Supporting Information

**Table S1:** Group size, sex ratio, and the steepness and linearity of the dominance hierarchy, together with their significance, for each group. Groups indicated in bold were used for the analysis considering the age-effect on rank. NA indicates that only one flee-upon-aggression was observed in this group, indicating that this group did not have a agonistic dominance hierarchy. ° P < 0.10; * P < 0.05; ** P < 0.01; *** P < 0.001.

| Group | Group size | Sex ratio | Steepness (P-value) | Linearity (P-value) |
| --- | --- | --- | --- | --- |
| GR01 | **6** | **0.50** | **0.328 (p = 0.022)*** | **0.979 (p = 0.035)*** |
| GR02 | 7 | 0.75 | 0.177 (p = 0.025)* | 0.441 (p = 0.263) |
| GR03 | 6 | 0.20 | NA | NA |
| GR04 | **6** | **1.00** | **0.438 (p < 0.001)***** | **0.858 (p = 0.092)°** |
| GR05 | 6 | 0.20 | 0.135 (p = 0.601) | 0.544 (p = 0.339) |
| GR06 | 10 | 0.67 | 0.326 (p < 0.001)*** | 0.429 (p = 0.169) |
| GR07 | 5 | 1.50 | 0.550 (p = 0.002)** | 0.993 (p = 0.178) |
| GR08 | **6** | **1.00** | **0.559 (p < 0.001)***** | **0.858 (p = 0.078)°** |
| GR09 | 9 | 0.50 | 0.298 (p < 0.001)*** | 0.519 (p = 0.133) |
| GR10 | 6 | 0.50 | 0.077 (p < 0.001)*** | 0.309 (p = 0.302) |
| GR11 | 6 | 1.00 |  |  |
| GR12 | **7** | **0.40** | **0.401 (p < 0.001)***** | **0.732 (p = 0.070)°** |
| GR13 | 5 | 0.67 | 0.450 (p = 0.004)** | 0.851 (p = 0.241) |
| GR14 | 5 | 0.25 | 0.266 (p = 0.100) | 0.798 (p = 0.285) |
| GR15 | 3 | 0.50 | 0.368 (p = 0.251) | 1.000 (p = 0.750) |
| GR16 | 4 | 1.00 | 0.236 (p = 0.244) | 0.895 (p = 0.398) |
| GR17 | 5 | 0.25 | 0.216 (p = 0.165) | 1.000 (p = 0.584) |
| GR18 | 5 | 0.25 | 0.278 (p < 0.001)*** | 0.524 (p = 0.222) |
| GR19 | 3 | 2.00 |  |  |
| GR20 | **15** | **0.50** | **0.254 (p < 0.001)***** | **0.578 (p < 0.001)***** |
| GR21 | 6 | 1.00 | 0.240 (p = 0.260) | 0.778 (p = 0.272) |
| GR22 | 5 | 1.50 | 0.550 (p < 0.001)*** | 0.603 (p = 0.497) |

**Table S2**: Correlation coefficients and corresponding p-values obtained after testing for collinearity among the different social network measures using Pearson’s correlations. Upper part of the matrix contains the correlation coefficients, the lower part contains the corresponding p-values. None of the correlation coefficients exceeded the threshold of |r| > 0.70. Significant correlations are indicated in bold. * P < 0.05; ** P < 0.01; *** P < 0.001.

|  | **Out-strength** | **In-strength** | **Disparity** | **Affinity** | **Eigenvector centrality** |
| --- | --- | --- | --- | --- | --- |
| **Out-strength** |  | **0.575 ***** | -0.023 | **0.628***** | **0.564 ***** |
| **In-strength** | < 0.001 |  | -0.013 | 0.082 | **0.580 ***** |
| **Disparity** | 0.795 | 0.884 |  | 0.052 | **0.196 *** |
| **Affinity** | < 0.001 | 0.345 | 0.551 |  | 0.057 |
| **Eigenvector centrality** | < 0.001 | < 0.001 | 0.022 | 0.509 |  |

**Table S3**: Post-hoc pairwise comparisons among the different sex-rearing history classes for in-strength. P-values were Tukey-adjusted. * P < 0.05; ** P < 0.01; *** P < 0.001.

| **Category** | **Estimate ± SE** | **Df** | **t-value** | **P-value** |
| --- | --- | --- | --- | --- |
| Female Atypical - Male Atypical | 0.046 ± 0.016 | 116 | 2.854 | 0.026* |
| Female Atypical - Female Mother | -0.004 ± 0.013 | 129 | -0.294 | 0.991 |
| Female Atypical - Male Mother | -0.006 ± 0.014 | 128 | -0.395 | 0.979 |
| Male Atypical - Female Mother | -0.050 ± 0.014 | 128 | -3.461 | 0.004** |
| Male Atypical - Male Mother | -0.051 ± 0.015 | 127 | -3.410 | 0.005** |
| Female Mother - Male Mother | -0.002 ± 0.009 | 117 | -0.191 | 0.998 |

**Table S4**: Detailed test statistics of the variables in the best fitting model according to the Akaike Information Criterium, for the five models testing the relationship between social network measures and individual and group-level characteristics. The reference categories for sex and rearing history were “Female” and “Mother-reared” respectively. * P < 0.05; ** P < 0.01; *** P < 0.001.

| Response variable | AIC | Predictor | Estimate ± SE | 95% CI | Df | t-value | P-value |  |
| --- | --- | --- | --- | --- | --- | --- | --- | --- |
| Out-strength | -455.8 | Sex | -0.006 ± 0.008 | -0.020 – 0.009 | 116.8 | -0.747 | 0.456 | |
|  |  | Age | 0.002 ± 0.000 | 0.001 – 0.003 | 117.7 | 3.664 | <0.001*** | |
|  |  | Age² | -0.478 x 10^-4^ ± 0.159 x 10^-4^ | -0.786 x 10^-4^ – -0.171 x 10^-4^ | 123.0 | -3.005 | 0.003** | |
|  |  | Rearing | 0.027 ± 0.011 | 0.006 – 0.048 | 129.6 | 2.488 | 0.014* | |
|  |  | Sex : Age | -0.002 ± 0.001 | -0.004 – -0.001 | 128.3 | -2.911 | 0.004** | |
| In-strength | -452.3 | Sex | -0.046 ± 0.016 | -0.081 – -0.012 | 116.0 | -2.866 | 0.005** | |
|  |  | Age | 0.001 ± 0.000 | 0.431 x 10^-3^ – 2.200 x 10^-3^ | 118.3 | 2.757 | 0.007** | |
|  |  | Age² | -0.371 x 10^-4^ ± 0.152 x 10^-4^ | -0.756 x 10^-4^ – -0.093 x 10^-4^ | 125.9 | -2.432 | 0.016* | |
|  |  | Rearing history | 0.004 ± 0.013 | -0.025 – 0.033 | 128.8 | 0.298 | 0.766 | |
|  |  | Sex : Rearing | 0.047 ± 0.018 | 0.010 – 0.089 | 116.1 | 2.613 | 0.010* | |
|  | -232.4 | Age | -0.003 ± 0.001 | -0.005 – -0.001 | 78.3 | -3.515 | 0.001** | |
| Disparity |  | Age² | 0.116 x 10^-3^ ± 0.037 x 10^-3^ | 0.040 x 10^-3^ – 0.189 x 10^-3^ | 74.7 | 3.108 | 0.003** | |
|  |  | Group size | -0.025 ± 0.008 | -0.041 – -0.010 | 17.1 | -3.120 | 0.006** | |
| Affinity | -455.16 | Sex | 0.028 ± 0.009 | 0.010 – 0.045 | 57.0 | 2.983 | 0.004** | |
|  |  | Age | -0.199 ± 0.421 | -0.001 – 0.001 | 65.8 | -0.473 | 0.638 | |
|  |  | Age² | 0.090 x 10^-4^ ± 0.154 x 10^-4^ | -0.208 x 10^-4^ – 0.383 x 10^-4^ | 57.6 | 0.583 | 0.562 | |
|  |  | Sex : Age | -0.490 x 10^-3^ ± 0.766 x 10^-3^ | -0.002 – 0.001 | 83.5 | -0.640 | 0.524 | |
|  |  | Sex : Age² | -0.103 x 10^-3^ ± 0.054 x 10^-3^ | -0.206 x 10^-4^ – 0.015 x 10^-4^ | 62.2 | -1.917 | 0.060 | |
| Eigenvector centrality | -117.5 | Sex | -0.188 ± 0.072 | -0.324 – -0.048 | 91.2 | -2.604 | 0.011* | |
|  |  | Age | 0.007 ± 0.002 | 0.004 – 0.011 | 85.6 | 3.813 | < 0.001*** | |
|  |  | Age² | -0.143 x 10^-3^ ± 0.060 x 10^-3^ | -0.255 x 10^-3^ – -0.030 x 10^-3^ | 63.7 | -2.393 | 0.020* | |
|  |  | Rearing history | 0.102 ± 0.039 | 0.028 – 0.174 | 88.8 | 2.636 | 0.010* | |
|  |  | Group size | -0.030 ± 0.005 | -0.039 – -0.020 | 114.8 | -5.798 | < 0.001*** | |
|  |  | Sex : Age | -0.005 ± 0.003 | -0.010 – 0.001 | 78.7 | -1.627 | 0.108 | |
|  |  | Sex : Group size | 0.023 ± 0.009 | 0.006 – 0.039 | 112.5 | 2.608 | 0.010* | |

**Table S5**: Detailed test statistics of the variables in the best fitting model according to the Akaike Information Criterium, for the models with disparity and eigenvector centrality as response variable, after standardizing them for group size (see “Materials and Methods” for standardization methodology). The reference categories for sex and rearing history were “Female” and “Mother-reared” respectively. * P < 0.05; ** P < 0.01; *** P < 0.001.

| Response variable | AIC | Predictor | Estimate ± SE | 95% CI | Df | t-value | P-value |
| --- | --- | --- | --- | --- | --- | --- | --- |
| Disparity | -237.5 | Age | -0.003 ± 0.001 | -0.005 – -0.001 | 79.2 | -3.552 | < 0.001*** |
|  |  | Age² | 0.115 x 10^-3^ ± 0.037 x 10^-3^ | 0.040 x 10^-3^ – 0.189 x 10^-3^ | 76.1 | 3.094 | 0.003** |
| Eigenvector centrality | 40.2 | Sex | -0.0275 ± 0.055 | -0.131 – 0.077 | 52.7 | -0.503 | 0.617 |
|  |  | Age | 0.014 ± 0.003 | 0.007 – 0.021 | 72.1 | 4.121 | < 0.001*** |
|  |  | Age² | -0.260 x 10^-3^ ± 0.109 x 10^-3^ | - 0.464 x 10^-3^ – -0.051 x 10^-3^ | 54.6 | -2.386 | 0.021* |
|  |  | Rearing history | 0.191 ± 0.073 | 0.048 – 0.330 | 86.3 | 2.598 | 0.011* |
|  |  | Sex : Age | -0.011 ± 0.005 | -0.021 - -0.002 x 10^-2^ | 76.6 | -1.927 | 0.056 |


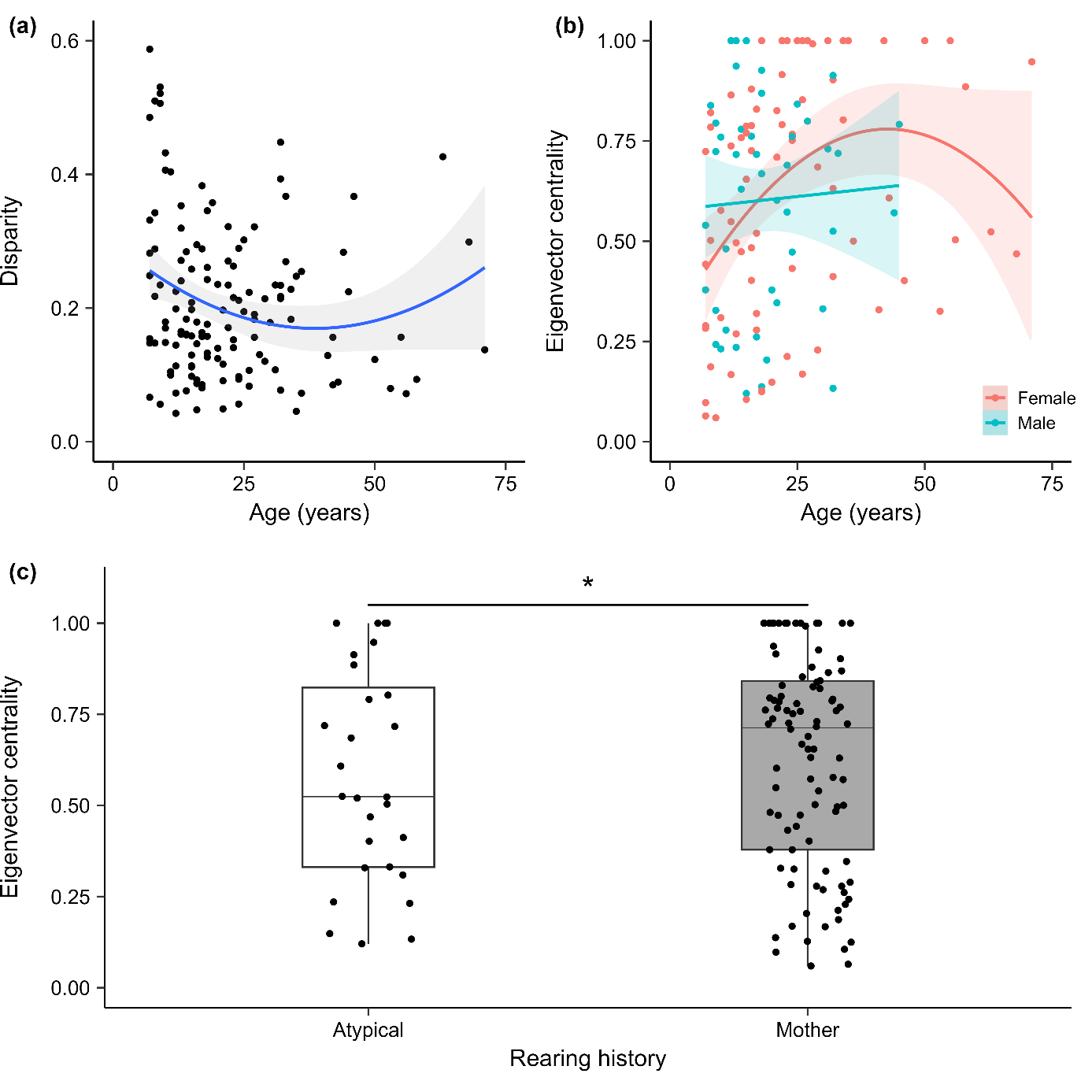


**Figure S1:** The influence of age on (a) disparity and (b) eigenvector centrality, with both measures corrected for group size. Males are indicated in blue datapoints with a blue trendline, while females are indicated in red. Shaded area represents the 95% confidence interval.
