# Supplementary material for: Multi-group analysis of grooming network position in a highly social primate
Source: PLoS One. 2023 Apr 26;18(4):e0284361. doi: 10.1371/journal.pone.0284361 (PMC10132689; doi:10.1371/journal.pone.0284361)
Supplement: Attachment — Submitted filename: Review of multi-group bonobo sna.docx [file pone.0284361.s003.docx]

**Review of *“Multi-group social network analysis provides comprehensive insights into bonobo grooming behavior”***

This study examines the relationships between various social network metrics and individual- and group-level traits in bonobos. Overall the manuscript is well-written and logically presented. The findings recapitulate some well-known results, but also extend to a number of novel results. The results will likely be of interest to primatologists interested in social behaviour as well as animal social network practitioners more generally. I have relatively few comments on the manuscript and would be happy to see it published with these changes.

**Comments:**

Title: The word “comprehensive” in the title suggests that nearly all aspects of bonobo grooming behaviour are examined. I think this is a stretch, and I suspect not exactly what the authors intended. Perhaps they would consider editing the title slightly?

L235: Recent work has shown that permutations don’t account for non-independence in this way (Hart et al., 2022). It doesn’t seem to affect results, so you could just change the justification of using permutations to “as per convention” or something like that.

L245: Very small terminology point - you refer to the distribution of coefficients as a posterior distribution, but since this is a frequentist analysis and is framed as repeated draws of a random variable, it is typically termed a *sampling distribution*.

L247: Stepwise regression has been critiqued as not respecting a causal model, and potentially leading to inflation of false positives (Smith, 2018). It might be useful to revisit those models, ensure the causal interpretation aligns with your predictions, and if not, rerun appropriate models.

L248 - 250: Could this be rephrased to make it clear that the permutation-based sampling distribution is not representative for small numbers of individuals, hence those small groups were excluded. I think this is point you’re trying to get across, but I struggled to understand what was being said a bit here.

L263 - 264: Adjusting alpha values to include near-significant effects is not necessarily best practice, as a fixed alpha value guarantees a given false positive rate under the null. A focus on confidence/credible intervals might be a better way to go for more nuanced analyses.

L275: Many of the effect sizes in the results sections are reported as being very small (around 0.0001). I assume the scale of the response and predictors is driving this, but perhaps some rescaling could be used to help readers understand the biological implications of these findings.

L438 - 439: I agree that multi-group analyses are important for generalisability, but I don’t quite find any hard evidence in this paper for why that is the case. Perhaps you could tone this message down a bit to make sure this type of statement is fully supported by the findings.

**References**

Hart, J. D., Weiss, M. N., Brent, L. J., & Franks, D. W. (2022). Common permutation methods in animal social network analysis do not control for non-independence. Behavioral ecology and sociobiology, 76(11), 1-10.

Smith, G. (2018). Step away from stepwise. Journal of Big Data, 5(1), 1-12.
