# Supplementary material for: Multi-group analysis of grooming network position in a highly social primate
Source: PLoS One. 2023 Apr 26;18(4):e0284361. doi: 10.1371/journal.pone.0284361 (PMC10132689; doi:10.1371/journal.pone.0284361)
Supplement: Attachment — Submitted filename: Response to reviewers.docx [file pone.0284361.s004.docx]

# Response to comments of the editor

Dear Dr. Torfs,

Thank you for submitting your manuscript to PLOS ONE. After careful consideration, we feel that it has merit but does not fully meet PLOS ONE’s publication criteria as it currently stands. Therefore, we invite you to submit a revised version of the manuscript that addresses the points raised during the review process.

Please submit your revised manuscript by Jan 26 2023 11:59PM. If you will need more time than this to complete your revisions, please reply to this message or contact the journal office at [plosone@plos.org](mailto:plosone@plos.org). When you're ready to submit your revision, log on to <https://www.editorialmanager.com/pone/> and select the 'Submissions Needing Revision' folder to locate your manuscript file.

Please include the following items when submitting your revised manuscript:

- A rebuttal letter that responds to each point raised by the academic editor and reviewer(s). You should upload this letter as a separate file labeled 'Response to Reviewers'.
- A marked-up copy of your manuscript that highlights changes made to the original version. You should upload this as a separate file labeled 'Revised Manuscript with Track Changes'.
- An unmarked version of your revised paper without tracked changes. You should upload this as a separate file labeled 'Manuscript'.

If you would like to make changes to your financial disclosure, please include your updated statement in your cover letter. Guidelines for resubmitting your figure files are available below the reviewer comments at the end of this letter.

If applicable, we recommend that you deposit your laboratory protocols in protocols.io to enhance the reproducibility of your results. Protocols.io assigns your protocol its own identifier (DOI) so that it can be cited independently in the future. For instructions see: <https://journals.plos.org/plosone/s/submission-guidelines#loc-laboratory-protocols>.

Additionally, PLOS ONE offers an option for publishing peer-reviewed Lab Protocol articles, which describe protocols hosted on protocols.io. Read more information on sharing protocols at <https://plos.org/protocols?utm_medium=editorialemail&utm_source=authorletters&utm_campaign=protocols>.

We look forward to receiving your revised manuscript.

Kind regards,

Daniel Redhead

Academic Editor

PLOS ONE

Journal Requirements:

When submitting your revision, we need you to address these additional requirements.

1. Please ensure that your manuscript meets PLOS ONE's style requirements, including those for file naming. The PLOS ONE style templates can be found at
   <https://journals.plos.org/plosone/s/file?id=wjVg/PLOSOne_formatting_sample_main_body.pdf> and 
   https://journals.plos.org/plosone/s/file?id=ba62/PLOSOne_formatting_sample_title_authors_affiliations.pdf

2. Your ethics statement should only appear in the Methods section of your manuscript. If your ethics statement is written in any section besides the Methods, please move it to the Methods section and delete it from any other section. Please ensure that your ethics statement is included in your manuscript, as the ethics statement entered into the online submission form will not be published alongside your manuscript.

We have revised the PLOS ONE style requirements. Our ethics statement has been provided in the Methods section.

Additional Editor Comments:

Two experts in the field, and myself, have now read the manuscript. All of us enjoyed reading the manuscript; it is well-written, clearly structured and speaks to research not only in primatology, but in social network analysis more generally. We all see great potential in the manuscript, with interesting findings among captive bonobos showing that an individual's position within a social network is patterned by age and gender. Both reviewers have flagged some serious issues with the statistical approach applied to analyse the complex data presented in the manuscript. Although Reviewer 2 suggests minor revisions, I must agree with Reviewer 1 as I think major revisions to the analytical strategy are necessary to ensure that reliable conclusions being drawn. The reviews are comprehensive and clearly detail all concerns. I wil not re-state everything here, but will outline the core points that I believe should be addressed during revision:

- Reviewer 1 notes that the many predictions/hypotheses outlined in the introduction should be unpacked and justified in greater detail.
- Use of the many associated centrality metrics should be clearly justified, and potentially re-thought
- Both reviewers have queried the use of permutations, and this analytical choice should either be clearly justified or re-considered.
- Both reviewers question whether the analysis approach closely reflects the data. Reviewer 1 goes further to softly suggest more principled, state-of-the-art analytical strategies (e.g., Hart et al., 2022, Ross et al., 2022), of which I believe would certainly strengthen the manuscript.
- Both reviewers ask for greater clarification of the present analyses and results related to multi-group analysis.

We are pleased that the editor enjoyed the manuscript. We have followed the suggestions by all reviewers and the editor and re-analyzed the data, which resulted in minor changes of our results. We have rewritten the paper accordingly. We have also worked on all other major points, as indicated in our comments below.

Important references to consider for revision:

Hart, J. D., Weiss, M. N., Franks, D. W., & Brent, L. J. (2021). Bison: A bayesian framework for inference of social networks. bioRxiv.

Hart, J. D., Weiss, M. N., Brent, L. J., & Franks, D. W. (2022). Common permutation methods in animal social network analysis do not control for non-independence. Behavioral ecology and sociobiology, 76(11), 1-10.

Franks, D. W., Weiss, M. N., Silk, M. J., Perryman, R. J., & Croft, D. P. (2021). Calculating effect sizes in animal social network analysis. Methods in Ecology and Evolution, 12(1), 33-41.

Hobson, E. A., Silk, M. J., Fefferman, N. H., Larremore, D. B., Rombach, P., Shai, S., & Pinter‐Wollman, N. (2021). A guide to choosing and implementing reference models for social network analysis. Biological Reviews, 96(6), 2716-2734.

# Response to Reviewer 1

Reviewer #1: I enjoyed reading this paper which presented some interesting data on affiliative networks in captive bonobos. The research reveals some interesting patterns relating sex and age to social network position within these captive groups, and it is great to see this relationship examined across multiple groups of different sizes and composed of different combinations of males and females, and old and young. I thought the framing of the paper in the introduction and discussion was excellent overall. That being said, I am a little concerned by some of the statistical approaches used and while I think there is a good chance these won’t have a substantial affect on the conclusions drawn, it would be good to see more robust methods applied. Thus I have indicated “No” as a response to related questions in the reviewer assessment, although am not a huge fan of these binary indications, as I feel that relatively small adjustments can address many of them.

We are pleased that Reviewer 1 enjoyed our paper and we thank them for their appreciation and helpful suggestions on how to improve the quality of our manuscript. To address the concerns raised by the two reviewers and the general editor regarding the statistical analysis, we made substantial changes to our analytical approach, stepping away from the node-based permutation tests. The methodological change has led to minor changes to the conclusions drawn and we have rewritten the paper accordingly.

It would also be really great to see more than just a summary dataset shared here – in my reviewer guide it states “For example, in addition to summary statistics, the data points behind means, medians and variance measures should be available.” And this is not the case with the data shared here, where it could be possible to share edge lists or networks to improve the replicability of these analyses.

Thank you for pointing this out. To allow for more detailed replication of our results we have now added the adjacency matrices with the grooming rates to the supplementary materials (S1 File).

One comment on the introduction is that I found the final two paragraphs providing the hypotheses/predictions rather challenging as a “naïve” reader. There are a lot of predictions packed in here and often the rationale for them isn’t especially clear – it hasn’t featured obviously in the introduction and then the explanation comes after the prediction. It may be helpful to play with the structure here to help readers have a good idea of how everything fits together (the table is definitely a big help and more could be made of this).

We have now expanded our hypothesis section to include a more broad introduction into bonobo grooming behavior. This way, all the necessary rationale and explanations come first, and the final two paragraphs of the introduction are mainly dedicated to listing our different hypotheses.

Building on this, part of the challenge is generated by using five broadly overlapping measures of social centrality, and there are a few related issues here beyond issues with the complexity of the story and elevated risk of false positive results (the latter of which I think is important for the authors to think about). First, how correlated are these different centrality measures in the bonobo networks studied? If some are closely correlated then it perhaps makes them more redundant and exacerbates risks associated with multiple testing. Second, it wasn’t clear to me what affinity would add to the other measures included here as a summary statistic of second order connectivity – it seems the insights here would be explained by any discrepancy between results from strength and eigenvector centrality results. Third, given the small size of the networks studied (and VERY small size of some) I am concerned about the meaningfulness of the indirect measures of centrality (eigenvector centrality and affinity). For the latter in particular there would seem to be some real issues with non-independence of the values of this measure that make it challenging to interpret. But even for eigenvector centrality, what can it tell us in a network of only 3, 4, 5 individuals that strength doesn’t?

We tested for correlation among all social network measures as this could indeed have implications for the interpretation of our results. We found that, even though all network measures correlated with at least one other measure, as could be expected, none of the correlations showed an |r| > 0.70, which was suggested as a threshold by Webber et al. (2020). All social network measures were used in separate models, to avoid violations of statistical assumptions. We also believe that the inclusion of all different measures aids in a more complete understanding of bonobo grooming networks, as they all represent different aspects of grooming behavior. For example, when considering grooming strength, we separated in- from out-strength because we know that grooming is used as a currency for exchange with other commodities like access to food, sex, etc. If we were to combine them, we can no longer make a distinction between grooming efforts made or received. The second reason for us to include so many variables is because some variables represent traditional measures, which allow for comparison with published literature for validation purposes (strength, disparity), while others have not been investigated in so much detail in bonobos and thus allow for novel insights and contributions to current grooming literature (eigenvector centrality, affinity). We have now added a paragraph to the methods section discussing this matter.

L236: “Prior to further analysis, we tested to what extent the grooming network measures correlated with each other. Correlations among different network measures could be high as these are all calculated from the same object (i.e., the grooming matrix), which excludes the use of different network measures in the same model and can influence the interpretation of the results of different models using the social network measure as a response variable [68]. Following the suggestions from Webber et al. [68], we a priori decided to exclude network measures if they showed a correlation coefficient with an absolute value above 0.70 with other network measures. Correlations among all five grooming network measures were assessed using Pearson’s rank correlations in R. We found that all network measures were correlated significantly with at least one other network measure (S2 Table), with the strongest correlation found between out-strength and affinity (r = -0.63, p < 0.001). We decided to investigate all five social network measures further in this study for three reasons: (1) the absolute values of all correlation coefficients were lower than 0.70, (2) all network measures will be used in different models, avoiding violation of statistical assumptions, and (3) each network measure represents a separate aspect of grooming behavior, and therefore, studying the different measures represents a more complete overview of bonobo grooming behavior which will aid in interpreting the results. This also allows for comparison of SNA measures with traditional grooming variables published in literature for validation purposes (such as strength and disparity), while other SNA measures have not been investigated in so much detail in bonobos and thus allow for novel insights and contributions to current grooming literature (such as eigenvector centrality and affinity).”

We agree with the reviewer that in small groups, indirect social network measures might not be highly informative. Still, we believe that the inclusion of the smaller groups is relevant to our study, as it allows for comparison with other studies on similar group sizes, and as it allows for a large variation in group sizes to test for effects of group size on social network position. We have now added a paragraph to the discussion about this matter. In an early stage of this study, we also ran the analysis excluding groups that had less than 5 individuals. This did not have any effect on the results, but since SNA studies on great apes sometimes include groups of 3 or 4 individuals, we decided to include all groups in the final study.

L507: “One important limitation of our study are the small sizes of some of the networks, which could influence indirect social network measures such as eigenvector centrality and affinity. In small networks, these measures are not that informative as these small networks usually show high densities with connections among most, if not all, dyads within the group. However, small networks are inherent to the study of SNA in zoo-housed great apes, as in previous work, group sizes typically ranged from 3 to 14 individuals [15,22,23,58,59]. Therefore, by including networks ranging from 3 to 15 individuals as in our study, we span the variation seen in previous studies which allows for direct comparisons. Moreover, the large range in group sizes allows for broad testing of group size effects on social network position.”

Given that the networks vary considerably both in size and sampling intensity (the range in the number of scan samples is sizeable) it would also be good for the authors to consider analytical options that address uncertainty in edge weights and account for this in subsequent analyses - e.g. <https://www.biorxiv.org/content/10.1101/2021.12.20.473541v1> - given this is the state-of-the-art this is a softer suggestion but think it would be particularly valuable to work such as this where greater weight could be placed on results from better sampled networks. Related to this, while I am relatively neutral to the use of permutations in animal social network analysis – in this particular case it is likely that not much is gained from using this approach (see <https://doi.org/10.1007/s00265-022-03254-x>). However, if the authors stick to using their permutation approach then there are a few additional considerations they need to take care with. First, the choice of permutation approach needs to be carefully justified – it strikes me that the node swaps as structured here work well for the individual-level questions (although perhaps could/should be restricted within networks or networks of similarly sized groups perhaps?) but that the justification for using this approach for the group-level analyses is much less clear. Second, I would strongly advise against combining the permutation approach with subsequent model selection – I would stick to using inference from the full model. Third, it is not consistent to use permutation methods to assess statistical significance because of concerns about non-independence and its affect on standard errors but then use confidence intervals when plotting figures – if permutation tests are the way to go perhaps these should be avoided.

We agree with the reviewer that the use of permutations in social network analysis is debated, with no clear consensus being reached at the moment. Upon suggestion of the reviewer we re-analyzed our data using parametric regressions following Hart et al. 2022. This has resulted in minor changes in results and we have re-written the paper accordingly.

To postulate upon the first point of Reviewer 1, our current data analysis protocol should be robust against differences in sampling intensity. To process our data, we used the “Index Of Interactions” approach, which was proposed by Sosa et al. (2021). As shown in this preprint, the index of interactions actually is robust against observation bias when combined with parametric regression and with permutations, as it returns low rates of false negatives and false positives, even in the presence of observation bias. We have added this to the method section.

L221: “Since the total observation time differed among groups, the grooming matrix was standardized [11] using the ‘index of interactions’ approach [65].”

I am also concerned about the structure of the data, and whether the use of random intercepts for individual and group are sufficient to control for this. In particular the fact that some groups are almost identical but differ in one or two members alone would seem likely to cause additional sources of non-independence, especially for relational data, i.e. the different groups are not fully independent of each other. It seems a really tricky dataset, and I’m not quite sure how best to suggest dealing with these types of issue (if they do indeed have the potential to lead to biased results). Regardless, I think more care going into the random effect structure of the model might be important here.

We agree with the reviewer that not all groups are fully independent of each other, as some of them share certain group members. However, there were only few groups reconsidered where only 1 individual differed between groups (N=3). We had extensive debates about this issue among coauthors but could not reach a consensus on how to better model this using our dataset as every solution that was offered came with its own limitations. For example, one could argue we set up the random structure by nesting ID (individual) into group-period (e.g., Planckendael 2012) into zoo (e.g., Planckendael), to account for the fact that some individuals were observed multiple times in the same group. However, this would not fully reflect the correct structure of group membership in our study population, as some individuals might be in the same zoo but not in the same subgroup (some zoos house multiple separate groups), and as there is also variation among individuals in how long they have been in a group together with other individuals. Also, some individuals might have lived together with certain individuals, got split up, and were then reunited again in another zoo. Such individual experiences are difficult to properly model and cannot be reflected in such a way for a model using datapoints on an individual level. If we were working with dyadic grooming rates, we could add these shared past experiences into the model, but for now, we were not able to do this and acknowledge this limitation in our discussion. We also know from experience that the addition of just one additional female or male (depending on their age and familiarity with the group members) can cause significant disruption within a stable grooming network, which is why we have opted for our current analytical approach. We are also happy to include any suggestions from the reviewer on how to better model these random effects.

L496: “In our analysis, we considered groups that differed in the presence or absence of at least one group member as fully separate networks, as this could have considerable effects on network structure [22,54] and thus their networks could be considered as independent. Still, different groups with a large proportion of the same group members might not be fully independent of each other, as tenure is known to influence social bonds in bonobos, with stronger social relationships found in individuals that have spent more time together [34,53]. Thus, we might expect that groups sharing certain group members might not be fully independent of each other. However, it is difficult to control for this in our models as we modelled our data on an individual basis. Future studies should look into how variation in dyadic tenures among individual group members structure social networks as a whole, and what the effect of introducing new animals or removing certain group members is on the structure of the social network of the group.”

With regard to the group-level analyses, I have several aspects I am confused about. First, given group size is included in the individual-level model it is not immediately clear why a separate analysis for group size is required (albeit the model is more simple). Second (related to the first point), given that group size was already included in the individual-level model and so any inference from (at least the full model) already was with respect to group size effects then it is not clear to me what repeating the analysis after standardising for group size (L320-323) achieves in addition, assuming that the correction is linear? These corrections are also inadequately described (how did you correct), nor are they included in the methods. Finally, with the interpretation of the group size results, this seems to me like it could be non-intuitive, as there may be background (null) relationships between network measure values and group size that depend on aspects of the generative model underlying network structure (e.g. how network density scales with group size). This remains unknown in animal societies but it may be worth reflecting on when discussing these group-level results.

Since some factors showed significant group size effects in the individual analysis, we simply wanted to test whether any of these associations with sex, age, and rearing history remained after correcting for group sizes or were simply due to group size differences. Our methods for standardization were added to the supplementary materials in the previous version but now we have added a paragraph in the methods section to explain our rationale behind it and to explain how the analysis was performed.

L280: “If an effect of group size was found on a certain network measure, we re-ran the model of this network measure after standardizing it for group size, to test whether any other associations still remained and whether they were independent of the effect of group size. To standardize in-strength and out-strength, we divided the measure by the number of available grooming partners (i.e. N-1, with N being the group size) [7]. Disparity was standardized by calculating the ‘deviation from edge weight disparity’, following the methodology of Kalcher-Sommersguter et al. [23]. Eigenvector centrality and affinity were standardized by scaling the value of each individual to the maximum value within their network [54]. Thus, the maximum in each network has a value of 1 and all others values are scaled towards this value. Subsequent analyses were performed as described above with separate models for each network measure, except that group size was removed as a predictor from all models.”

We have also added the points brought up by the reviewer to our discussion. We now found group size effects on eigenvector centrality and disparity, and especially for eigenvector centrality, these might be the result of scaling effects of the networks. However, in the case of eigenvector centrality, the effect of group size differed between the sexes which implicates that additional social effects are at work.

I would also encourage the authors to explore the impact of captivity and the captive environments in their discussion a little more – this does come up (especially in regard to the movement of individuals in a way that mirrors dispersal in the wild), but it feels like there may be other aspects to this. For example, how does group size correspond to the size/complexity of the enclosure and could this impact social structure/affiliative interactions at all? Is there anecdotal evidence of individuals occupying similar positions in different networks or moving individuals causing substantial changes in network structure. I don’t think these make/break interpretations of the results, but they were some questions that arise when thinking about comparing different captive groups.

This is a good point brought up by the reviewer that we only touched upon lightly in the original manuscript. We have now added a paragraph to the discussion highlighting this matter.

L516: “Ideally, our analyses should be repeated in wild groups of bonobos, as certain aspects of the captive environment might influence grooming rates. For example, we did not account for density (i.e., number of individuals per surface area in the enclosure), even though higher densities could lead to increases in grooming rates [89,90]. In the wild, group size will be independent of available space, and as such, grooming rates and network structure will not depend on any density effects and the social implications of group size could be investigated more reliably.”

Line-by-line comments:

L24-25: It would be great if a clearer indication of the direction of results was given here and the two parts of the sentence either separated or more clearly linked with each other.

We expanded the abstract such that we could give more attention to the results.

L23: “Our results showed age-effects on all investigated measures: for females, all measures except for affinity showed quadratic relationships with age, while in males, the effects of age were more variable depending on the network measure. Bonobos with atypical rearing histories showed lower out-strength, while in-strength was only impacted by rearing history in males. Group size showed a negative association with disparity and eigenvector centrality, while sex ratio did not influence any of the investigated measures.”

L40-42: Try and avoid the circularity that arises from the second “network” here – currently the sentence reads as social network analysis is a great tool for analysing social networks, where if the wording was changed it could read that SNA is a great tool for studying social systems and the positions of individuals within them more generally.

We changed the wording accordingly.

L41: “Social network analysis (SNA) provides a useful toolkit to study social systems and an individual’s social position within the group, as it offers an opportunity to quantify various measures that represent different aspects of these direct and indirect relationships [2,7,8].”

L44: Missing citation for the point about longevity

We added the citation.

L49: I’m not a primatologist. Is it accurate to say that social bonds are maintained by grooming relationships only or would a more qualified statement be clearer here to avoid potential confusion?

We have rephrased this sentence since social bonds do not solely depend on grooming relationships, but grooming relationships are a good proxy for social bonds and are often used to study sociality in primates.

L50: “In primates, the time spent grooming others is often used as a proxy for the strength of their social bonds [13,14].”

L61: Would suggest providing a more careful and accurate definition of eigenvector centrality. The one here is at odds with the way the eigenvector centrality is often used as a measure and its accuracy will depend on the structure of the network (the one used in the table is much better).

We rephrased this sentence to include a more accurate definition of eigenvector centrality.

L62: “eigenvector centrality represents an individual’s embeddedness within the entire grooming network as it is a measure for how well an individual is associated, as well as how well their connections are associated themselves”

L113-115: This sentence doesn’t make any sense as it is currently written – I think there might be an error which leads to one part seeming to say the opposite to the other?

This sentence indeed contained an error. It has been rewritten correctly.

L137: “We predict males will have higher affinity compared to females, because males tend to prefer females as grooming partners [40–43,45], and usually occupy the lowest ranks in the hierarchy and might therefore focus their grooming on high-ranking individuals that are expected to have high strengths [31,47].”

L120: Again, this may be as a result of my naivety about primatology, but this relies on assumptions about post-reproductive lifespan that haven’t been introduced from the literature yet. Are these post-reproductive lifespans only found in captivity?

We have now added a statement about the age span of the reproductive period in bonobos.

L115: “thus grooming activity is expected to be highest during the time-frame of reproduction, which in female bonobos lies roughly between 6 and 40 years old [49].”

L137-141: This hypothesis makes a set of strong assumptions about whether in- and out- interactions are mutually exclusive (presumably not for grooming interactions? You can groom another individual while being groomed yourself?) and the latter part of the argument seems tenuous for strength – I can see the case for increased disparity in large groups but not how this leads to reduced mean in-strength? For example, if one individual spends 60% time of its time being groomed and the rest of individuals no time, then this is the same mean strength as all 6 individuals being groomed for 10% of the time. It strikes me that this argument alone is insufficient to explain differences in mean in-strength.

We agree with the reviewer that our hypotheses regarding the link between in-strength and group size needed reconsideration. We have adjusted our hypotheses to say that in-strength shows no changes with group size, but will rather be dependent on individual ‘popularity’ and social attractiveness, which in turn depends on individual characteristics like sex, age, and rearing history.

L152: “Group size is not expected to impact in-strength as the amount of grooming received will rather depend on individual characteristics such as sex, age, and rearing history.”

L165-166: The infants were not included in the networks or not included in the analysis? This is unclear currently.

Juveniles were not included in the networks, as we had no detailed data on these individuals for all observation periods. This is stated more clearly now.

L181: “All groups consisted of individuals of both sexes, and some groups also contained juveniles and/or infants (i.e., individuals under 7 years old). Juveniles and infants were not included in the networks as we did not collect reliable data on their grooming behavior.”

L171-173: Suggest changing “Given that” to “Because” as initially I misunderstood this sentence because of this initial wording

We changed the wording accordingly:

L187: “Because some individuals were transferred between groups over time, and in some institutions groups were observed multiple times over the 11 year time-span, certain individuals were observed more than once.”

L178: It is not clear what this reliability score refers to? It is a little concerning if it is a reliability of individual identity for a network study given the impacts this could have on the observed network structure (although perhaps this is the norm in primatology)

This inter-observer reliability score refers to the similarity between observers in observing and coding the correct behaviors shown in the videos using our standardized ethogram. Small differences are typically found between observers in the speed of coding which result in slightly divergent durations and frequencies of mostly fast-occurring behaviors like scratching. This thus does not have anything to do with individual recognition of animals. All observers go through an extensive training period and are required to be able to identify each individual without error before they can start their data collection.

Table 3: Would suggest providing this information by individual as well as by data point.

We now provide the information by individual between brackets in the same table.

L216-217: Eigenvector centrality was calculated using the weighted networks?

Indeed, all measures were calculated using the weighted network matrices. We have now stated this more clearly.

L232: “All measures were calculated from the weighted networks.”

L225-226: It’s not entirely clear to me why the correction is only relevant here?

Since we changed our analytic approach, the original sentence was deleted as we could now use specialized R-packages to perform post-hoc comparisons.

L235-237: The rationale of using within-group swaps for the questions tackled here is not clearly explained. It makes sense to me (I think) for the individual-based questions, but doesn’t seem like a relevant permutation test for the group-level questions. L246: I don’t think this is correcting the p-value so much as calculating a new p-value using a different approach. L248-251: I find these two sentences very unclearly explained and confusing.

As we have changed our analytical approach, these original sentences are now deleted from the manuscript.

Figure 3: How much leverage is there of eigenvector centrality measures from very small groups, especially given the measure isn’t especially meaningful in these very small groups?

Their leverage is minimal as there are only a few datapoints available from these small groups. We also added a paragraph to the discussion about the caveats associated with small groups (see above).

L320-323: How have you conducted these standardisations and why is this not in the methods?

A paragraph has now been added to the methods section (see also above).

L417: Presumably this could also be driven simply by the rules linking group size and network density without invoking any more complex patterns?

This might indeed be the case. We also based our predictions on these rules. We have now more explicitly stated this again in our discussion. Still, it remains interesting that the effect differs between males and females, suggesting that certain sex-differences in sociality might influence the response of eigenvector centrality to group size, so we still kept this in the discussion.

L474: “In line with our expectations, eigenvector centrality significantly decreased with group size. Larger groups have less dense and more modular networks [28], leading to lower eigenvector centralities for individual group members. However, it is interesting that the effect differed between the sexes, with a larger effect in females than in males. Females show a greater reduction in eigenvector centrality with group size than males do, which could be explained by differences in social patterns between the sexes. Females will form strong grooming bonds with all group members, while male bonobos are more selective in their grooming bonds and tend to prefer high-strength female group members to groom with. Hence, when group size increases and groups become more female-biased in our sample, females will more drastically drop in centrality as more equally central females are present, while males have more high-strength females to associate with, thereby showing a less drastic reduction in centrality. Moreover, the finding that group size effects on eigenvector centrality differ between males and females illustrate why multi-group studies are important. As seen in Fig 3B, in smaller groups, females tend to have higher eigenvector centrality than males, while in the larger groups, males seem to have higher eigenvector centrality than females. If only one group is studied, a sex-difference in eigenvector centrality might actually simply be a consequence of the group size and might not reflect an absolute difference in social embeddedness between the sexes.”

L427: How do you propose this is done? It is incredibly challenging, especially when trying to tease apart true biological effects from network effects

Based on research in previous publications (e.g., Balasubramaniam et al., 2021; Crailsheim et al., 2020; Kalcher-Sommersguter et al., 2015; Kasper & Voelkl, 2009), we have standardized our measures and re-run the analysis. We have now added our methods and the rationale behind it more clearly in the methods section of the manuscript (see above).

I hope my comments help improve the paper.

We would like to thank this reviewer again for their careful review of our paper and helpful comments for improvement of our manuscript.

# Response to Reviewer 2

Reviewer #2: Review of “Multi-group social network analysis provides comprehensive insights into bonobo grooming behavior”

This study examines the relationships between various social network metrics and individual- and group-level traits in bonobos. Overall the manuscript is well-written and logically presented. The findings recapitulate some well-known results, but also extend to a number of novel results. The results will likely be of interest to primatologists interested in social behaviour as well as animal social network practitioners more generally. I have relatively few comments on the manuscript and would be happy to see it published with these changes.

We thank the reviewer for their kind and helpful feedback on our manuscript. Based on the feedback from this reviewer and also from reviewer 1 we have decided to opt for a different statistical approach which hopefully alleviated most of the concerns raised below.

Comments:

Title: The word “comprehensive” in the title suggests that nearly all aspects of bonobo grooming behaviour are examined. I think this is a stretch, and I suspect not exactly what the authors intended. Perhaps they would consider editing the title slightly?

We changed the title to: “Multi-group analysis of grooming networks in a highly social primate”.

L235: Recent work has shown that permutations don’t account for non-independence in this way (Hart et al., 2022). It doesn’t seem to affect results, so you could just change the justification of using permutations to “as per convention” or something like that.

We did decide to change our analysis to parametric regression, as suggested also by Reviewer 1. It indeed only had minor effects on the results and final conclusions.

L245: Very small terminology point - you refer to the distribution of coefficients as a posterior distribution, but since this is a frequentist analysis and is framed as repeated draws of a random variable, it is typically termed a sampling distribution. L248 - 250: Could this be rephrased to make it clear that the permutation-based sampling distribution is not representative for small numbers of individuals, hence those small groups were excluded. I think this is point you’re trying to get across, but I struggled to understand what was being said a bit here.

As we have changed our analytical approach, the original sentences were not relevant anymore and are now deleted from the manuscript.

L247: Stepwise regression has been critiqued as not respecting a causal model, and potentially leading to inflation of false positives (Smith, 2018). It might be useful to revisit those models, ensure the causal interpretation aligns with your predictions, and if not, rerun appropriate models.

Due to changes in our analytical approach we now select the best-fitting model using AIC.

L263 - 264: Adjusting alpha values to include near-significant effects is not necessarily best practice, as a fixed alpha value guarantees a given false positive rate under the null. A focus on confidence/credible intervals might be a better way to go for more nuanced analyses.

While we agree with the reviewer on this comment, it is unfortunately not possible to work with confidence intervals when analyzing linearity of our dominance data using the available software as it only returns the value of linearity and a p-value based on permutations. While David’s scores are the preferred method for determining dominance and are in our opinion more reliable than, for example, the use of keeper interpretations of dominance (which are also commonly used in literature), they have the limitation that they require data on submissive interactions within the majority of dyads in the group to produce significant results. However, in reality, submissive behavior and aggression are rare in our populations and definitely less frequent and even sometimes absent in female-female dyads. With higher uncertainties (e.g., dyads with no interactions) in the flee-upon-aggression matrix, it is difficult to obtain a significant linear dominance hierarchy because some dyads will occupy similar rank positions. However, if we were to apply a more stringent approach (e.g., alpha set at 0.05), we would have only 2 groups left which does not allow for a representation of sex-specific rank correlations with age. With this limitation in mind we decided not to include our rank results into our SNA models, but rather use them as a visual aid to help interpret the age-related changes in grooming.

L275: Many of the effect sizes in the results sections are reported as being very small (around 0.0001). I assume the scale of the response and predictors is driving this, but perhaps some rescaling could be used to help readers understand the biological implications of these findings.

These small effect sizes are indeed the consequence of the scale of the response variables and predictors. We chose not to rescale our variables as our data is easy to interpret in terms of percentages, e.g. out-strength refers to the frequency of grooming given, with an out-strength of 0.15 on a graph indicating that this individual groomed 15% of the time during observations.

L438 - 439: I agree that multi-group analyses are important for generalisability, but I don’t quite find any hard evidence in this paper for why that is the case. Perhaps you could tone this message down a bit to make sure this type of statement is fully supported by the findings.

Thank you for pointing this out. While we did not explicitly mention this in great detail in the manuscript, our findings regarding the sex-group size interaction on eigenvector centrality offer a clear example of the importance of multi-group studies. When comparing sex-effects on eigenvector centrality in the different groups on fig 3B, we see that in smaller groups it is the females who have higher centrality whereas in larger groups males have a tendency towards higher centrality. If any single group would have been studied alone, the large variation and opposite effects based on group size would not be detected. We have added this to the discussion.

L484: “Moreover, the finding that group size effects on eigenvector centrality differ between males and females illustrate why multi-group studies are important. As seen in Fig 3B, in smaller groups, females tend to have higher eigenvector centrality than males, while in the larger groups, males seem to have higher eigenvector centrality than females. If only one group is studied, a sex-difference in eigenvector centrality might actually simply be a consequence of the group size and might not reflect an absolute difference in social embeddedness between the sexes.”

References
Hart, J. D., Weiss, M. N., Brent, L. J., & Franks, D. W. (2022). Common permutation methods in animal social network analysis do not control for non-independence. Behavioral ecology and sociobiology, 76(11), 1-10.

Smith, G. (2018). Step away from stepwise. Journal of Big Data, 5(1), 1-12.
