# Supplementary material for: Multi-group analysis of grooming network position in a highly social primate
Source: PLoS One. 2023 Apr 26;18(4):e0284361. doi: 10.1371/journal.pone.0284361 (PMC10132689; doi:10.1371/journal.pone.0284361)
Supplement: Attachment — Submitted filename: review.docx [file pone.0284361.s005.docx]

I’m pleased to see this manuscript again, and I’m glad the authors have made many of the changes suggested by the reviewers. I feel the manuscript is now much improved. I am now mostly happy with the manuscript, though I still have two outstanding concerns.

Firstly, I thank the authors for their response regarding my comment about the scale of the predictors, and how that might affect the interpretability of the regression coefficients. The authors note that they feel the scale is appropriate because it allows coefficients to be interpreted as changes in % time spent. I agree with the authors in theory, but in practice, a coefficient value of e.g. β = -0.002 (from L331) is difficult to interpret biologically. Perhaps the authors could also provide a short explanation of what this means biologically, for example, an difference in age of 10 years would be associated with a difference in out-strength of around 2%. This might help readers to appreciate the biological magnitude of these effects.

A related point is that the authors were unable to report confidence intervals for their models. I strongly feel reporting intervals helps to biologically ground the results, so that readers appreciate the scientific implications of the findings. Confidence intervals can usually be extracted from most popular software packages. I understand lmerTest runs tests on lme4 models, and lme4 supports the computation of confidence intervals. I would encourage the authors to revisit this point and see if intervals could be reported. I feel it would really help readers to appreciate these interesting findings.

I’m looking forward to seeing the paper published after these changes are made.
