# Supplementary material for: Multi-group analysis of grooming network position in a highly social primate
Source: PLoS One. 2023 Apr 26;18(4):e0284361. doi: 10.1371/journal.pone.0284361 (PMC10132689; doi:10.1371/journal.pone.0284361)
Supplement: Attachment — Submitted filename: Response to reviewers.docx [file pone.0284361.s006.docx]

# Response to comments of the editor

Dear Dr. Torfs,

Thank you for submitting your manuscript to PLOS ONE. After careful consideration, we feel that it has merit but does not fully meet PLOS ONE’s publication criteria as it currently stands. Therefore, we invite you to submit a revised version of the manuscript that addresses the points raised during the review process.

Both reviewers and I are pleased to see many of our suggested changes have been made, and believe that these changes have greatly strengthened the manuscript. For this reason, I suggest only minor changes need to be made to the manuscript to make it suitable for publication. That being said, Reviewer 1 raises several important points about the analyses presented in the manuscript, which should be addressed in detail in your revision.  These comments are detailed below.

Please submit your revised manuscript by Apr 26 2023 11:59PM. If you will need more time than this to complete your revisions, please reply to this message or contact the journal office at [plosone@plos.org](mailto:plosone@plos.org). When you're ready to submit your revision, log on to https://www.editorialmanager.com/pone/ and select the 'Submissions Needing Revision' folder to locate your manuscript file.

Please include the following items when submitting your revised manuscript:

- A rebuttal letter that responds to each point raised by the academic editor and reviewer(s). You should upload this letter as a separate file labeled 'Response to Reviewers'.
- A marked-up copy of your manuscript that highlights changes made to the original version. You should upload this as a separate file labeled 'Revised Manuscript with Track Changes'.
- An unmarked version of your revised paper without tracked changes. You should upload this as a separate file labeled 'Manuscript'.

If you would like to make changes to your financial disclosure, please include your updated statement in your cover letter. Guidelines for resubmitting your figure files are available below the reviewer comments at the end of this letter.

If applicable, we recommend that you deposit your laboratory protocols in protocols.io to enhance the reproducibility of your results. Protocols.io assigns your protocol its own identifier (DOI) so that it can be cited independently in the future. For instructions see: <https://journals.plos.org/plosone/s/submission-guidelines#loc-laboratory-protocols>. Additionally, PLOS ONE offers an option for publishing peer-reviewed Lab Protocol articles, which describe protocols hosted on protocols.io. Read more information on sharing protocols at <https://plos.org/protocols?utm_medium=editorial-email&utm_source=authorletters&utm_campaign=protocols>.

We look forward to receiving your revised manuscript.

Kind regards,

Daniel Redhead

Academic Editor
PLOS ONE

Journal Requirements:

Please review your reference list to ensure that it is complete and correct. If you have cited papers that have been retracted, please include the rationale for doing so in the manuscript text, or remove these references and replace them with relevant current references. Any changes to the reference list should be mentioned in the rebuttal letter that accompanies your revised manuscript. If you need to cite a retracted article, indicate the article’s retracted status in the References list and also include a citation and full reference for the retraction notice.

We have reviewed the reference list. No retracted papers were included in our study.

Additional Editor Comments (if provided):

The manuscript is reading much better now, and I have slightly more confidence in the analyses that are presented. I agree with Reviewer 1 on several core concerns that remain in the current manuscript:

- The use of indirect metrics of centrality in small networks. Is it meaningful to interpret such a metric such as eigenvector centrality on a network of such a size (e.g., 3 nodes/individuals)? Given how such metrics are calculated, I would suggest that it is not and that only analyses of direct measures should be included.
- Why did the authors not opt for more state-of-the-art approaches  (e.g., using a social relations model) highlighted by Reviewer 1 and myself? This analytical choice should be at least acknowledged and justified in the manuscript.
- Please rethink and at least better justify the use of David scores.
- Please include confidence intervals for all relevant results and further respond to Reviewer 2's points about interpretation of coefficients.

We are pleased that the editor enjoyed this new version of the manuscript. We have followed the suggestions of all reviewers and the editor and included responses to each concern in more detail below. We would like to thank the editor again for their helpful comments on this manuscript, which greatly improved the study as a whole.

# Response to Reviewer 1

Reviewer #1: In general, I feel the authors have done a great job addressing concerns from the reviewers, and the paper is now clear, well-written and well-explained.

We are pleased that Reviewer 1 enjoyed the new version of our manuscript.

I have to admit that I remain unconvinced about the wisdom of using indirect measures in the smallest networks presented here. I appreciate the authors have provided a detailed caveat in the discussion, and perhaps this is sufficient. However, this remains problematic if the measures used have different biological interpretations in these very small networks. It is unclear to me what variation in eigenvector centrality would mean in such small networks, and I am concerned that variation in affinity in small networks represents a different trait to its variation in larger networks given that in networks of 3 individuals the least connected individual will almost certainly have the highest affinity which is not true in larger networks (e.g. in toy small networks with 3 nodes, affinity often seems highest for the least integrated individual). Given the results seem consistent when the smallest networks are excluded (mentioned by the authors in their response), I am happy to leave it to the discretion of the editor as to how important this concern is.

To circumvent the issues related to indirect network measures in small groups, we have limited our analyses of affinity and eigenvector centrality to groups with 5 or more individuals, only slightly reducing the size of the dataset compared to the direct measures (10 individuals from 3 groups were removed for the analyses of indirect measures). We chose this threshold of a group size of 5 to remove the smallest groups from our dataset but also minimize the number of groups that had to be removed, such that enough power remained to test for group-level effects on the indirect measures. We decided to re-analyze affinity and eigenvector centrality in our manuscript, rather them remove them completely, as these are the most novel within the bonobo literature, since only two small single-group studies have previously reported on eigenvector centrality and none have reported on affinity. Hence, we believe that it is of interest to maintain these indirect grooming network measures in the manuscript, and we hope that we have solved the concerns of Reviewer 1 and the editor by running the analyses on a smaller dataset excluding these small groups. For affinity, the results remained the same as in the previous version of the manuscript. For eigenvector centrality, rearing-history was a fixed effect in the remaining best model whereas in the full dataset this was not the case. Parent-reared bonobos are more central in the network compared to other-reared bonobos, which was one of our hypotheses. We have rewritten our results and discussion section accordingly. We hope that our re-analysis resolved the concerns raised by Reviewer 1 and the editor.

It is also a little disappointing to see the authors not try some of the more state-of-the-art statistical approaches highlighted by the editor, although I think that given the nature of the sampling here (fairly comprehensive and relatively even across groups), the current approach is probably adequate, and I wouldn’t expect the results to change substantially. I do think the current approach selected has improved the clarity of the paper considerably. (A quick side note for the authors to consider for their future research. While the index of interactions approach is probably OK in this case given the intensity of data collection and relative ease with which individuals can be observed, it is not accurate to say that it deals as well with variation in sampling intensity as newer approaches. Through being an index, individuals observed grooming each other 1 out of 2 times is recorded in the same way as individuals recorded grooming each other 100 out of 200 times (index of 0.5). However, there is a huge difference in the amount of information these two sets of observations carry about the social relationship between two individuals, which is why approaches such as the BISoN framework recommended previously, and other similar approaches are preferable in general)

We apologize for not meeting some of the expectations of the Reviewer, but we were under the impression that it mostly concerned the removal of our permutation approach, which we implemented in the novel version of the manuscript. We were reluctant to implement the BISoN package at this stage of our research as it is, to our knowledge, currently only available as a non-peer reviewed preprint and has not been used in other SNA studies. Given the current finalized state of this paper, we were therefore hoping to include the BISoN package in a follow-up study where we want to link SNA data to physiological measures. We have contacted the author of the package with a request for assistance with these analyses since we are running into some issues regarding certain limitations of the bisonR package. We hope the reviewer and editor both agree that the field of SNA is a rapidly evolving one with lots of opposing views regarding methodological approaches, but we hope that given the size and data collection protocol of our dataset the current analytical approach is considered sufficient and offers comparable results to what is currently the norm for published primate SNA research.

We have added a paragraph to the discussion where we discuss the usefulness of novel techniques in improving the reliability of social network analyses, hereby highlighting their existence and potential for application in future SNA studies.

L537-550: “As the study of animal social networks is a rapidly developing field, especially regarding advances in statistical methodology, future studies on primate grooming networks could aim to include more state-of-the-art analytical approaches to investigate variation in grooming network measures. For example, besides using parametric models or permutation methods, generative models can be used to study social network data as well, as they account for the non-independence of social network data [89]. In addition, as sampling effort varies across groups, but also within groups (e.g., certain dyads might be observed more often than others and hence their grooming relationship might be more ‘certain’), uncertainty in network edges could be taken into account in downstream analyses. Instead of correcting for variation in sampling effort using commonly used social network indices, using other frameworks such as the newly developed BISoN framework [90] could provide a way to incorporate sampling effort and edge uncertainty into down-stream analyses. In our current study, sampling effort was relatively even across groups and across dyads within the groups due to the high visibility of individuals, reducing edge uncertainty. Nonetheless, future studies could take advantage of these newly developing analytical techniques to more reliably investigate primate social networks.”

I am also not fully convinced by how the authors have used the AIC approach here for model selection. First, I’m not sure it fully addresses the other reviewer’s concerns related to the statistical modelling (which seemed more aimed at pushing for a causal understanding, and would perhaps favour a well justified full model inference approach). Second, the mixture of AIC model selection (which weighs the support for the model as a whole) and F tests that test the importance of single variables within the model seems rather inconsistent.

We are not entirely sure we understand what the reviewer means with this remark. Based on the comment of the other reviewer we now apply AIC as a model selection approach rather than a backward model selection approach and then report the variables included in this best model with their estimates and significance in the model. We did make an error on our end by reporting F-tests, since we did not run additional F-tests to determine significance of factors but rather rely on estimates and corresponding test statistics of the fixed effects and interactions (if any were present in the final model) from the model summaries implemented within the lmerTest package, which uses t-tests. We are happy to incorporate a different approach if needed, but would like more feedback on how to do so.

L275-278: “We first ran full models and then reduced the models based on the Akaike Information Criterium (AIC), such that the most parsimonious model with the lowest AIC value remained [72]. Model summaries were used to determine the parametric coefficients of the fixed effects in the remaining models.”

L311-318: As to some extent already highlighted by the authors, there are severe limitations to this analysis – the authors extract David scores without accounting for uncertainty around them, the complexity of the fixed effect structure (number of variables plus interactions) is high for a small sample size (n=40), and the number of random effect levels is also small (n=5) to accurately estimate random effect variance. I’m a little torn as the model results remain fairly convincing despite (or perhaps in the case of the uncertainty) because of these limitations, but equally I wonder whether the plot (Fig. 1F) is suitably convincing without needing to resort to using the statistical approach in the first place. Again, I am happy to leave this to the discretion of the editor/authors.

We agree that our analysis of David’s scores were not highly reliable, which is why we decided to not incorporate rank into the models but rather use it as a visual aid to explain the age-related patterns that many network measures follow in our study. As the rank analysis is not the focus of our study, there is no real need to run statistical analyses to model age effects on them so following the reviewers suggestions we removed them from the study and only included the visualization to support our discussion.

Otherwise, I have just a few very minor points.

L181-182: There is a typo in this sentence

The typo has been corrected:

L181-182: “Juveniles and infants were not included in the networks as we did not collect reliable data on their grooming behavior.”

L332-333: It is not clear to me how an “exponential” decline in affinity with age was detected when the relationship(s) tested were quadratic (and linear). I assume the authors were trying to draw attention to the fact that affinity only declined in older individuals, and the rate of decline accelerated with age (Fig. 1d)? On a related point, there seems to be a missing line/confidence interval Fig. 1d? It would also be good to position the legend (male/female colours) in such a way that it applies to all panels of the plot.
We indeed meant to refer to a quadratic relationship, rather than an exponential one. We have rewritten this sentence accordingly:

L337-339: “Affinity showed a tendency towards a quadratic relationship with age in male bonobos, with the rate of decline being shallow in younger bonobos, but accelerating with age, with the lowest values found in old male bonobos.”

We re-made the figures and placed a legend in each panel where sex-differences in age patterns were found. We also added the missing trendline in panel 1D.

L400-405: There are some potential links here with wider literature on social selectivity with age (e.g. https://doi.org/10.1073/pnas.2209180119 and the papers it cites).

Here, the reviewer refers to our finding that old male bonobos groom less than younger males (the same goes for females), as shown by a significant reduction in their out-strength. This does not necessarily reflect a change in social selectivity, as these males might still invest equally in different grooming partners, but might just show a general decrease in grooming efforts. But we included this suggestion later on, where we discuss disparity, given that disparity increases in older bonobos, which is in line with higher social selectivity. We have added this paper to the discussion there as we only referred to chimpanzee studies in the previous version of the manuscript, but not to studies investigating other primate species.

L445-453: “With age, disparity declined, but in old age, disparity increased again, showing bonobos become more selective again after their thirties. These results are in line with a recent body of literature reporting similar effects of higher social selectivity with age across a variety of primate species [e.g., 19,51,85].”

L450: Describing this as a “vast” majority seems excessive. It would also be good to point to the fact you only have 22 groups so have relatively low power at this level of the analysis.

We have rewritten our discussion here following these suggestions:

L471-481: “However, this result is to be treated with caution as the majority of groups in our study were female-biased (14/22 female-biased groups versus 3/22 being male-biased), and there was a confound between group size and sex ratio, with the largest groups in our study all being female-biased (S1 File and S1 Table). This is important to consider when interpreting the results, as for some network measures, opposite effects between group size and sex-ratio would be expected in large male-biased groups (Table 2), although these are rare in bonobos due to the lower life-expectancy of male bonobos [49,87]. Moreover, due to the relatively low power our study has on the group-level, a possible effect of sex ratio on grooming network position might have gone undetected. Still, it needs to be noted that a previous study did not find any effect of group sex ratio on grooming patterns in wild bonobos [88], so perhaps bonobo grooming strategies are relatively independent of the sex ratio of the group.”

L478-481: I find this explanation hard to understand as it rests on sex ratios influencing centrality measures, and you did not find a direct effect of these measures when they were included in your statistical models

We agree with the reviewer here that this sentence was not entirely clear. We have now rewritten the sentence as follows:

L498-505: “Females show a greater reduction in eigenvector centrality with group size than males do, which could be explained by differences in social patterns between the sexes. Females will form strong grooming bonds with all group members, while male bonobos are more selective in their grooming bonds and tend to prefer high-strength female group members to groom with. Hence, when group size increases and thus the absolute number of females in the group increases, females will more drastically drop in centrality as more equally central females are present, while males will have more high-strength females available to associate with, thereby showing a less drastic reduction in centrality.”

L502: effectS

We have corrected this typo:

L513: “As the effects of our individual level variables (sex and age) on grooming metrics remained identical after correction for group size, they appear to have a robust effect on grooming behavior that is independent of demographic variation.”

We would like to thank the reviewer for their helpful and constructive comments on the current and previous version of our manuscript.

# Response to Reviewer 2

I’m pleased to see this manuscript again, and I’m glad the authors have made many of the changes suggested by the reviewers. I feel the manuscript is now much improved. I am now mostly happy with the manuscript, though I still have two outstanding concerns.

We are pleased that Reviewer 2 enjoyed the new version of our manuscript and that they acknowledge the improvement of the study as a whole.

Firstly, I thank the authors for their response regarding my comment about the scale of the predictors, and how that might affect the interpretability of the regression coefficients. The authors note that they feel the scale is appropriate because it allows coefficients to be interpreted as changes in % time spent. I agree with the authors in theory, but in practice, a coefficient value of e.g. β = -0.002 (from L331) is difficult to interpret biologically. Perhaps the authors could also provide a short explanation of what this means biologically, for example, an difference in age of 10 years would be associated with a difference in out-strength of around 2%. This might help readers to appreciate the biological magnitude of these effects.

We understand why reviewer 2 raises this point, and following their suggestion, we have now added several sentences to the results section where we describe the age-related patterns in words. For quadratic relationships, the patterns are more difficult to put concisely into words as the increase and/or decrease in social network measures accelerates or decelerates when you move further or closer from the maximum or minimum. In these cases, we discussed in which age categories the maxima and minima of the social network measures fell. For significant linear effects, we discuss the changes with age in terms of percentages, as suggested by the reviewer. For example:

L332-335: “In males, out-strength declined linearly with age (Out-strength: β = -0.002 ± 0.001 SE, t(128.3) = -2.911, p = 0.004), indicating that for each 10-year increase in age, males decrease their time spent on grooming others with 2%. To put this in perspective, the average out-strength for males across all ages is 0.079, indicating that about 8% of their time is spent on grooming others.”

A related point is that the authors were unable to report confidence intervals for their models. I strongly feel reporting intervals helps to biologically ground the results, so that readers appreciate the scientific implications of the findings. Confidence intervals can usually be extracted from most popular software packages. I understand lmerTest runs tests on lme4 models, and lme4 supports the computation of confidence intervals. I would encourage the authors to revisit this point and see if intervals could be reported. I feel it would really help readers to appreciate these interesting findings.

This is a good point from the reviewer, as by an error on our side we forgot to report standard errors or confidence intervals in our results section and only included standard errors in the supplement. We have now added the standard errors of the estimates in our results section and we have added confidence intervals in the tables in the supplementary information, which contain all of the test statistics per model.

I’m looking forward to seeing the paper published after these changes are made.

We again would like to thank the reviewer for their constructive feedback on the current and previous version of the manuscript.
